# Human Illness from Avian Influenza H7N3, British Columbia

**DOI:** 10.3201/eid1012.040961

**Published:** 2004-12

**Authors:** S. Aleina Tweed, Danuta M. Skowronski, Samara T. David, Andrew Larder, Martin Petric, Wayne Lees, Yan Li, Jacqueline Katz, Mel Krajden, Raymond Tellier, Christine Halpert, Martin Hirst, Caroline Astell, David Lawrence, Annie Mak

**Affiliations:** * British Columbia Centre for Disease Control, Vancouver, British Columbia, Canada;; † Health Canada Field Epidemiology Training Program, Ottawa, Ontario, Canada;; ‡ Fraser Health Authority, Abbotsford, British Columbia, Canada;; § Canadian Food Inspection Agency, Ottawa, Ontario, Canada;; ¶ National Microbiology Laboratory, Winnipeg, Manitoba, Canada;; # Centers for Disease Control and Prevention, Atlanta, Georgia, USA;; ** Hospital for Sick Children, Toronto, Ontario, Canada;; †† British Columbia Cancer Agency Genome Sciences Centre, Vancouver, British Columbia, Canada

**Keywords:** influenza virus, avian, zoonoses, surveillance, epidemiology, human, chickens, dispatch

## Abstract

Avian influenza that infects poultry in close proximity to humans is a concern because of its pandemic potential. In 2004, an outbreak of highly pathogenic avian influenza H7N3 occurred in poultry in British Columbia, Canada. Surveillance identified two persons with confirmed avian influenza infection. Symptoms included conjunctivitis and mild influenzalike illness.

Influenza is the most diversified in birds, particularly in wild waterfowl (*1*). Concern exists that outbreaks of avian influenza in domestic poultry could, through a process of genetic reassortment, mutation, or both, introduce new influenza subtypes into the human population. In the context of widespread susceptibility, such an event could be the precursor of a pandemic (*2**,**3*).

An outbreak of avian influenza emerged on a farm in the Fraser Valley of British Columbia on February 6, 2004. Slightly increased deaths (8–16 deaths/day) were noted among 9,200 chickens in one barn. Avian influenza infection was confirmed on February 16, 2004, and later genotypic and phenotypic intravenous pathogenicity index (IVPI) testing characterized the virus as low pathogenicity avian influenza (LPAI) H7N3. On the same farm, an adjacent barn that contained 9,030 chickens had a dramatic increased in deaths from February 17 through 19 (2,000 deaths in 2 days). Genotypic and IVPI testing confirmed highly pathogenic avian influenza (HPAI) H7N3 in this second flock.

The Canadian Food Inspection Agency ordered the culling of both flocks and initiated active avian influenza surveillance on all farms within 5 km, but the virus spread nonetheless. On April 5, the Canadian Food Inspection Agency ordered depopulation of all poultry in the Fraser Valley south of the Fraser River (19 million birds). In total, the Canadian Food Inspection Agency identified avian influenza in 42 of the ≈600 commercial poultry farms in the region and in 11 backyard flocks, which represented ≈1.3 million birds (*4*). The last infected farm was identified on May 21, 2004.

To mitigate the risk for human infection and the potential for genetic reassortment, federal workers involved in the depopulation were required to wear personal protective equipment, including N95/North 7700 masks, gloves, goggles, and biosafety suits and footwear. They were also required to take prophylactic oseltamivir at a dose of 75 mg per day for the duration of exposure plus 7 days and to receive the commercially available human influenza vaccine for the 2003-04 season, if they had not already done so (*5*). All protective measures were provided free of charge and were recommended also for exposed farm workers and their families. Following reports of human illness, these measures were more rigorously promoted and reinforced through worker screening, information letters prepared by the British Columbia Centre for Disease Control, and media bulletins.

We report the results of enhanced surveillance for human illness in association with this poultry outbreak of HPAI H7N3 in British Columbia.

## The Study

After the first report to public health authorities of poultry outbreaks on February 18, 2004, enhanced surveillance for conjunctivitis and influenzalike illnesses was implemented for federal workers, farm workers and their household contacts, and any other potentially exposed persons. Illness was reported to the British Columbia Centre for Disease Control by using a standard questionnaire and report form. Respiratory specimens were tested at the British Columbia Centre for Disease Control by reverse transcription–polymerase chain reaction for influenza and by cell culture for all respiratory pathogens; influenza isolates were sequenced to determine the subtype (e.g., H7). Suspected human cases were defined as illness in persons presenting after February 6, 2004, with two or more new or worsening conjunctivitis or influenzalike symptoms, with onset from 1 day after first exposure (defined as direct contact or shared air space) to 7 days after last exposure to a potential source of avian influenza virus in the Fraser Valley. Confirmed cases had laboratory-confirmed influenza A (H7) virus in conjunctival, nasal, nasopharyngeal, or throat specimens by reverse transcription–polymerase chain reaction (*6*) or cell culture. Influenza hemagglutinin and neuraminidase subtyping was performed at the National Microbiology Laboratory. Serum samples were tested for antibody to influenza A (H7) by hemagglutination inhibition and microneutralization assays (*7*) at the National Microbiology Laboratory. Microneutralization assays were repeated at the U.S. Centers for Disease Control and Prevention on serum samples from two persons with confirmed infections and from eight persons with suspected cases.

Approximately 2,000 poultry farm workers are in the Fraser Valley. Approximately 650 federal workers assisted with outbreak management and control; not all had poultry exposure. From February 18 to June 1, 2004, a total of 77 symptomatic persons were reported to the British Columbia Centre for Disease Control. Fifty-seven had suspected (n = 55) or confirmed (n = 2) avian influenza infections.

Among the 20 reports that did not meet the suspected or confirmed case definitions, 9 had insufficient information to determine case status, 3 did not meet the symptom requirements, 3 did not have a relevant exposure history, 1 had onset before February 6, 3 had onset >7 days after exposure, and 1 had onset <1 day after exposure.

Baseline characteristics are shown in Table 1, and the epidemic curve is shown in the Figure. Respiratory symptoms predominated (Table 2) among the 55 patients with suspected cases. Symptom duration was 1–58 days. No patients were hospitalized. Twelve (22%) reported taking prophylactic oseltamivir at symptom onset, and 11 (20%) received oseltamivir for treatment. The remaining 22 patients with suspected cases were identified >48 hours after onset or refused treatment. All recovered fully.

**Table 1:** Characteristics of patients with suspected and confirmed A/H7N3 cases

Characteristic	Cases (%) (N = 57)
Male sex	32 (58)
Median age in y (range)	33 (1–68)
Received influenza vaccine	36 (65)
>2 wk before exposure	12 (22)
Occupation/relationship	
Farm owner	9 (16)
Family member	11 (19)
Farm employee	14 (25)
Farm manager	3
Egg collector	6
Chicken catcher	2
Miscellaneous worker	3
Federal worker	12 (22)
Veterinarian	4^a^
Inspector	3
General laborer	6^a^
Decomposition worker	1
Other	4 (7)
Unknown	5 (9)

^a^Includes confirmed A/H7N3 case.

**Figure:**
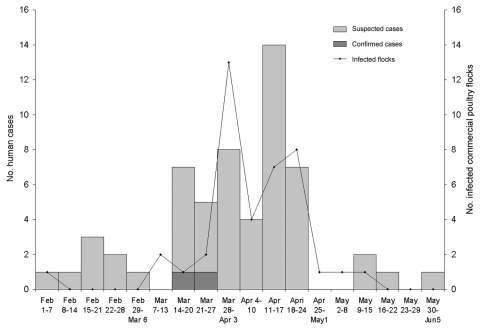
Onset of symptoms for suspected and confirmed cases in humans and identification of infected commercial poultry flocks, highly pathogenic avian influenza H7N3, British Columbia, 2004. Date for poultry flock is either the date the flock was suspected to be infected (because of clinical illness) or the date the sample was taken as part of surveillance.

**Table 2:** Clinical profile of suspected and confirmed infections of avian influenza H7N3 in humans, Canada

Symptoms	Cases (%) (N = 57)
Conjunctivitis	
Red eye	12 (21)^a^
Tearful eye	6 (11)^a^
Itching eye	13 (23)^a^
Painful eye	4 (7)
Burning eye	7 (12)
Discharge from eye	4 (7)^a^
Photophobia	5 (9)
ILI symptoms^b^	
Fever	17 (30)
Cough	42 (74)
Coryza	35 (61)^a^
Sore throat	39 (68)
Myalgia	26 (46)
Arthralgia	16 (28)
Fatigue	22 (39)
Diarrhea	11 (19)
Chills	16 (28)
Headache	28 (49)^a^
Other symptoms	12 (21)

^a^Includes confirmed A/H7N3 cases. ^b^ILI, Influenza-like illness.

Respiratory specimens (nasal, nasopharyngeal, throat, and conjunctival) were collected from 47 patients with suspected cases (86%) an average of 5 days after onset (range 0–27 days). Cell culture identified pathogens in two persons: adenovirus type 3 in one (conjunctival and nasal specimens) and HSV-1 in another (throat specimen). All other results were negative for respiratory viruses, including influenza. No antibody to influenza A H7 could be detected in paired acute- and convalescent-phase serum samples (n = 17), drawn an average of 9 days (range 0–33 days) and 31 days (range 18–88 days) after onset, respectively, or in convalescent-phase serum samples (n = 8) drawn an average of 28 days (range 8–56) after onset from patients with suspected cases.

Influenza A H7N3 infection was confirmed in two men (40 and 45 years of age) exposed on different farms March 13 and March 22–23. Both had direct conjunctival contact with infected poultry. One was not wearing eye protection, and the other was wearing glasses that were bypassed by a feather. Neither was taking oseltamivir prophylaxis. Neither was vaccinated against human influenza virus. Symptoms developed 1–3 days after exposure (March 16 and 24). Conjunctivitis and coryza developed in the first patient, and conjunctivitis and headache developed in the second. Both received oseltamivir treatment, and symptoms resolved fully. Active daily surveillance by the local health unit identified no secondary cases.

Influenza A H7N3 virus was isolated from a nasal specimen from one man (A/Canada/444/04) and a conjunctival specimen from the other (A/Canada/504/04); both samples were collected within 1 day of onset. No antibody to influenza A H7 could be detected by hemagglutination inhibition or microneutralization assays in serum samples collected 34 days and 8 and 22 days after onset, respectively.

Virus isolated from birds on the same source farm as the human isolate A/Canada/444/04 was confirmed as HPAI H7N3 by genotyping and IVPI. Virus from birds on the same source farm as A/Canada/504/04 showed insertion sequence match with HPAI H7N3, but IVPI was not performed (C. Kranendonk, National Centre for Foreign Animal Disease, pers. comm.). Both human isolates contained an insertion sequence similar to that seen only in the HPAI avian virus. These insertion sequences vary from the poultry virus by one and two amino acid differences, respectively. Based on the consensus sequence for HPAI H7 viruses, only A/Canada/504/04 is likely highly pathogenic in chickens (*8*). Phenotypic pathogenicity testing on the human isolates is ongoing.

## Conclusions

We report the first known human avian influenza H7N3 infections. Although enhanced surveillance identified 57 persons meeting a suspected case definition, avian influenza infection was confirmed in only 2. The two patients had conjunctivitis and mild, influenzalike illnesses, similar to symptoms reported from the Netherlands in association with another H7 subtype (H7N7) (*9*). Neither confirmed case in British Columbia mounted a hemagglutination inhibition or serum neutralizing antibody response. This finding has been observed elsewhere in association with avian influenza infection (*10**,**11*). A possible explanation includes highly localized infection without induction of systemic antibody. Mechanical trauma, irritation due to dust or airborne particulate matter, or an allergic cause of symptoms associated with viral contamination, rather than infection, is less likely given the delay to symptom onset, consistent with the incubation period for influenza.

Among suspected cases, respiratory rather than conjunctival symptoms predominated. Other pathogens were also detected among suspected case reports, a finding consistent with the relatively nonspecific case definition applied.

From February 6 to May 21, 2004, routine influenza surveillance activities in the Fraser Valley also identified human influenza A from nine persons and two long-term care facility outbreaks. Although no coinfections were identified, this human influenza activity increased concerns about potential mixture of avian influenza with human influenza strains.

Avian influenza H7 has caused human illness previously, most notably 89 confirmed human infections, including one death in the Netherlands in 2003 (*9*). Based on the precedent set by the Netherlands in protecting exposed persons, British Columbia recommended comprehensive precautions for workers early in the outbreak. These precautions may have prevented further human infections. The strain circulating in British Columbia may have been more limited in its ability to cause human illness. The genomic sequence of the avian viruses from the source farms of the two human isolates was consistent with HPAI, whereas one of the human isolates was consistent with LPAI. The presence of an insertion sequence in the human LPAI isolate likely signifies that the virus in poultry mutated from HPAI to LPAI, and both were circulating among the birds on that source farm, the latter undetected. A less likely explanation is that mutation from HPAI to LPAI occurred in the human host.

To date, illness in humans from H7 subtypes differs markedly in severity from that of avian influenza H5N1 (*12*). Their lower virulence should not be inferred to indicate lower pandemic potential since subclinical or mild infections may have greater opportunity through surreptitious spread to reassort and through mutation to become more virulent. A compilation and detailed overview of the protective measures used in all avian influenza outbreaks would help to estimate the actual risk to persons and populations. Recommendations for precautions that are both necessary and reasonable during future poultry outbreaks could then be refined.
